# The impacts of flowering phenology on the reproductive success of the narrow endemic *Nouelia insignis* Franch. (Asteraceae)

**DOI:** 10.1002/ece3.7747

**Published:** 2021-06-18

**Authors:** FangYan Liu, ChengJie Gao, Min Chen, GuoYong Tang, Yongyu Sun, Kun Li

**Affiliations:** ^1^ Research Institute of Resources Insects Chinese Academy of Forestry Kunming China; ^2^ Desert Ecosystem Research Station in Yuanmou County of Yunnan Province State Forestry Administration of China Yuanmou China; ^3^ College of Life Science Southwest Forestry University Kunming China

**Keywords:** dry and hot valley, Jinsha River, pollination, reproductive barrier, self‐incompatibility

## Abstract

*Nouelia insignis* Franch. (Asteraceae) is a short, narrow endemic and endangered tree, growing with a natural population in the dry and hot valley of the Jinsha River in the southwest area of China. In this work, flowering phenology (time and duration), floral biology, visit frequency and behavior of pollinators, and pollination characteristics were studied based on investigation in the field and analysis in the laboratory with the help of a stereomicroscope, and the relationship between seed setting rate and reproductive traits, as well as the relationship between flowering time and rainfall before flowering, was tested using the method of general linear regression model. The results showed that natural population of *N. insignis* exhibited high flowering synchrony with relatively stable flowering duration, and the flowering time fluctuated greatly depending on the rainfall 5 months before flowering. The pollination of *N. insignis* required pollinators, and insect activities played a very important role in the pollination process. However, lack of the pollinators was not a limitation for reproductive fitness in *N. insignis*, although the number of pollinators was small and the frequency of visits was low. In addition, no pollen limitation was found during pollination. The average seed setting rate of *N. insignis* in the natural condition was only 1.52%–3.73%, and it was generally affected by changes in flowering phenology between years and had a higher seed set in early flowering year. The annual variation of seed set might be related to the annual variations of stamen and pistil functions, such as changes of pollen viability and stigma receptivity, which were closely related to flowering time. The results of this study are of value for further conservation actions on natural population of this threatened endemic plant.

## Graphical abstract

Natural population of *Nouelia insignis* exhibited high flowering synchrony with relatively stable flowering duration, and the flowering time fluctuated greatly between years depending on the rainfall 5 months before flowering. The pollination of *N. insignis* required pollinators, and insect activities might play a very important role in the pollination process. However, lack of the pollinators was not a limitation for reproductive fitness in *N. insignis*. The annual variation of fruit set might be related to the annual variations of stamen and pistil functions, such as changes of pollen viability and stigma receptivity, which were closely related to flowering time.

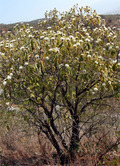

## 1 INTRODUCTION

Flowering is an important phase of plant life cycle, which strongly affects plant fitness (Hafdahl & Craig, 2014; Rathcke & Lacey, 1985; Sandring et al., 2007). Flowering phenology at the community level is often affected by several ecological factors, and the period during which flowering produces the maximum seed setting rate may vary from year to year, depending on the availability of resources (Mahoro, 2002; Rathcke & Lacey, 1985). For example, it is possible to observe different flowering phenology patterns for species in dry, harsh, and variable environments because of temporally limited water resources, and reproductive success usually depends on the time of onset of flowering (Cortés‐Flores et al., 2017; Tarayre et al., 2007), which can be related to plant strategies to access water sources deep in the soil (Borchert et al., 2004). Flower‐visiting insect is another factor affected by flowering time, constituting one of the most important interactions for seed production. This interaction may influence the evolution of flowering time through competition for pollinators, leading to the selection of asynchronous flowering, or promoting pollinators to improve service through synchronous flowering (Cortés‐Flores et al., 2017; Rathcke & Lacey, 1985). For example, in many seasonal dry forests, most canopy plants flower simultaneously during droughts (van Schaik et al., 1993), and attracting more pollinators and exposure to pollinators have been proposed to explain this pattern. Furthermore, changes in flowering time and synchronization have been associated with regularity and behavior patterns of insects' visits to flowers, and the activity patterns of insects are usually consistent with the flowering phenology of associated types of plants (De Jong & Klinkhamer, 1991; Fuchs et al., 2003; Hafdahl & Craig, 2014). Insect‐specific flowering plants must rely on the activity of pollinators to complete the ovule fertilization process, and temporal overlap with pollinators is an important factor in the evolution of flowering phenology (Tarasjev, 1997; Totland, 2001). Therefore, changes in the behavior patterns of flower‐visiting insects have a particularly serious impact on the reproduction of these plants (Aguirre & Dirzo, 2008). Exploring the relationship between flowering phenology and insect visitors and their behavior, seed production, and some ecological factors, as well as the degree of temporal and spatial variation in these relationships, can provide insight into the selection power that affects the evolution of flowering time.

Several features of the floral biology, such as pollen vitality and stigma receptivity, are particularly important in reproductive success of a population, and they were also affected by flowering time (Clivati et al., 2014; Gao et al., 2004; Hong et al., 2011; Thompson, 2001). Strong evidence has shown that there are significant differences in pollen viability, longevity, and stigma receptivity as well as the encounter periods of pollen and stigma among different flowering periods and habitats (Cui et al., 2008; Liu et al., ; Nebot et al., 2016; Nelizabeth & Sedoniad, 2010; Rymer et al., 2005; Wei & Huang, 2006). However, little information is available on the precise relations between those major components, or particular features as the relationship between pollen viability and stigma receptivity of the plant and flowering time, and the changes in pollen longevity and stigma receptivity through flowering time. Studies of the interaction between these factors are fundamental prerequisites for an understanding of the reproductive constraints which affect a given population.

**FIGURE 1:**
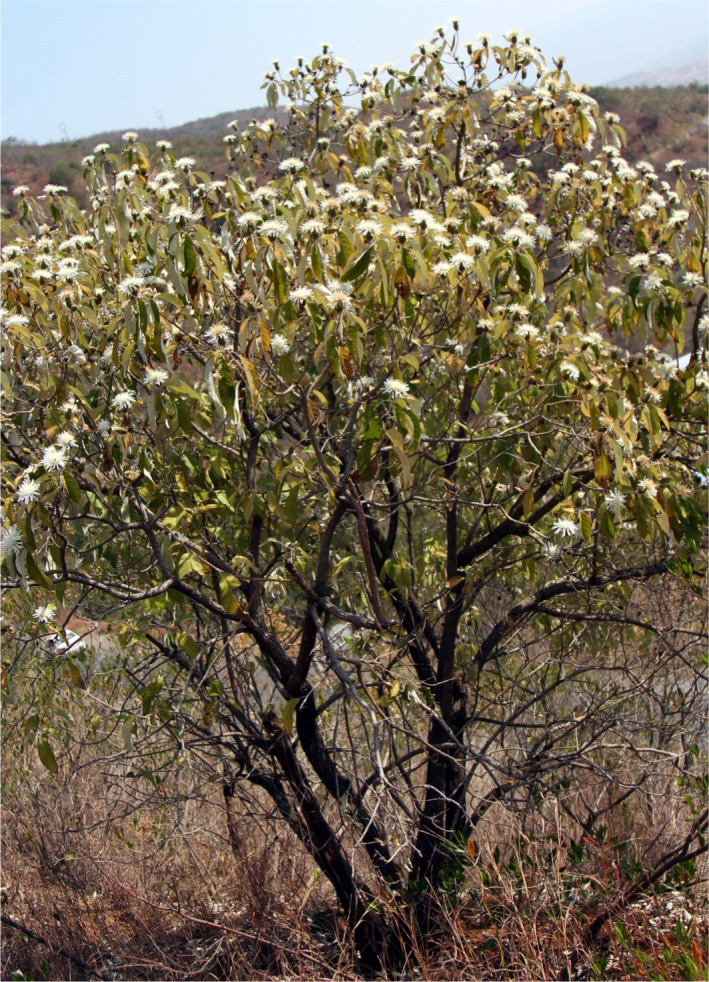
*Nouelia insignis* blooming in its natural environment

*Nouelia insignis* Franch. is a species of *Mutisieae* (Asteraceae), and it is characterized by an unusual woody growth form. *Nouelia insignis* is a small tree with abundant branches, a height of 3–5 m, and a diameter at breast height (DBH) of 10–20 cm (Figure 1). It grows in dry valleys within 1,000 to 2,800 m a. s. l. in the Jinsha and Nanpan drainage areas in southwestern China (Peng et al., 2003). *Nouelia insignis* has become endangered, and most of the populations are seriously threatened. The species suffers from reproductive failure because of low seed productivity and seed germination rates, especially along the Jinsha River drainage. Therefore, very few seedlings could be located in the natural habitats (Peng et al., 2003). According to field observations, all *N. insignis* populations are fragmented and patchy. These populations are mostly distributed in habitats with steep slopes and poorly developed soil (Gong et al., 2011). Some of populations are even on the brink of extinction. Most *N. insignis* populations do not exceed 80 individuals, and the total number of individuals in all populations does not exceed 5,000 (Luan et al., 2006). Regeneration failure of endangered *N. insignis* trees in the dry and hot valleys of southwestern China is an important ecological issue, which is attracting more attention than in the past. Studies have shown that inherent factors (e.g., lower fertility, lower viability, and lower adaptability) of endangered plants are fundamental drivers of their endangered status (Zhang et al., 2002). In recent years, research on the flowering phenology and flowering biology of rare and endangered plants has attracted significant amounts of attention. However, the relationship between flowering phenology and reproductive success of *N. insignis* have not yet been reported. This paper aims to provide new information about the reproductive biology of *N. insignis* grown in a dry and hot environment. The following aspects were investigated: (a) flowering phenology, (b) floral biology, (c) pollinators, and (d) rainfall before flowering. We hypothesized that *N. insignis* with different flowering phenology in different years will have differences in floral biology and behavior patterns of insects' visits to flowers, and the reproductive success of *N. insignis* is closely related to their flowering phenology, floral biology, the activities of its pollinators, and rainfall before flowering.

## 2 METHODS

### 2.1 Study site

This study was conducted in the dry‐hot valley of Jinsha River, Yuanmou, Yunnan, southwest China (25°40′N, 101°52′E; 1,200 m a. s. l.). The dry‐hot valley is characterized by dry and hot climate. Average annual rainfall (1991–2015) is 584 mm, with the biggest rainfall year (891 mm) occurs in 1998, and the driest year (471 mm) occurs in 2010. Rainfall distribution tends to be wet season dominant (June–October) with less than 10% of the annual rainfall falling in the dry season (November–May of the following year) when evaporative demand is highest. Annual potential evapotranspiration (1991–2015) averaged 3,848 mm. Thus, the trees grew in an environment where the atmospheric water demand was on average 6.6 times the rainfall. The long‐term average annual temperature was 22°C, and the lowest average monthly temperature was 15°C (December), with an extremely low temperature of −1°C, while the highest average monthly temperature was 27°C (May), with an extremely high temperature of 42°C. The annual accumulated temperature (≥10°C) was 8 003°C. The main type of soils is classified as Ferralic Arenosols according to the FAO Taxonomy (FAO‐UNESCO, 1988; Tang et al., 2013). There is shallow topsoil, and the subsoil layers are deep and compact and have a high level of gravel (mass fraction>35%) with poor water retention. Thus, the trees grew in an environment where there is a high degree of desertification and severe soil erosion, with ravines on the terrain surface.

### 2.2 Flowering characteristics and phenological records

**FIGURE 2:**
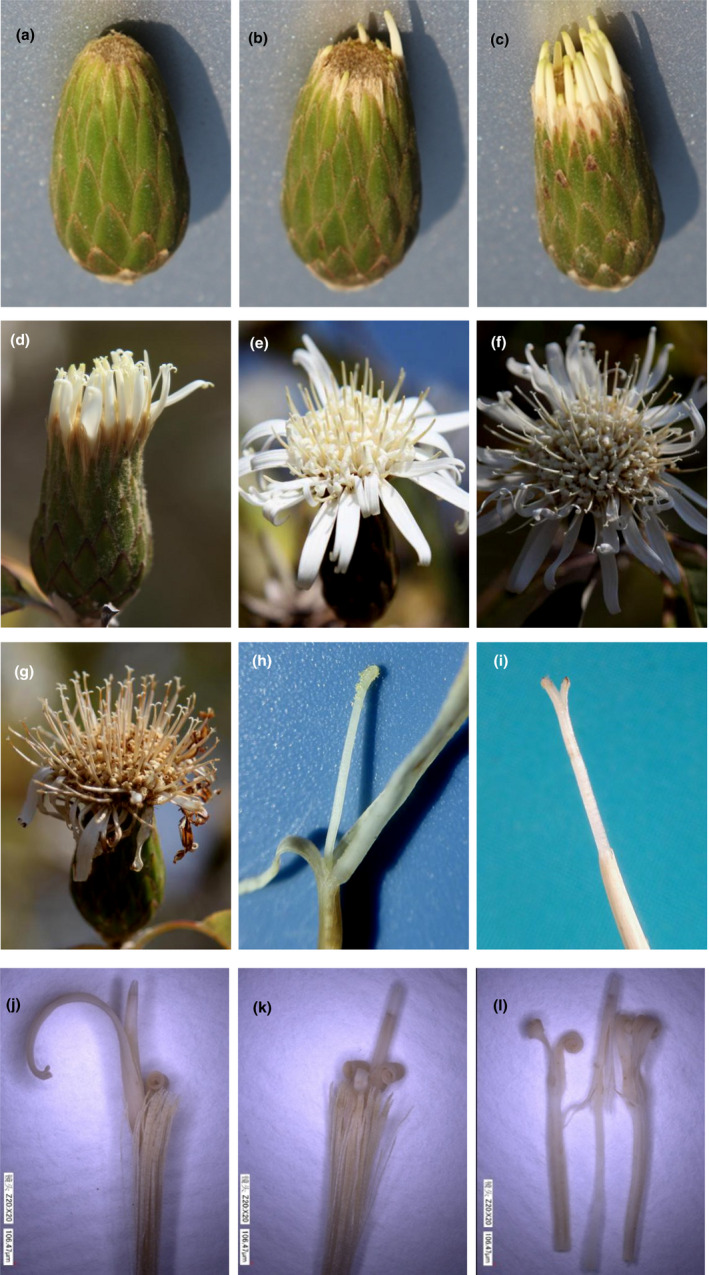
The floral morphological characteristics observed during flowering of *Nouelia insignis*. a–g: Morphological changes during flowering; h: The anther tube covered with pollen; i: the stigma sliver spread a “Y” type; j–l: Morphological structure of the florets

*Nouelia insignis* blooms from late February to early April. The capitulum is axillary or terminal on shoots with lengths of 4.1 ± 0.6 (mean ± *SD*) cm long. The maximum diameter is 1.8 ± 0.4 cm before flowers open and 5.2 ± 0.9 cm while flowering. The number of the florets in a capitulum is approximately 101 ± 12, including ray florets (marginal flower) (Figure 2j) and tubular florets (middle flower) (Figure 2k–l). When *N. insignis* blooms, the anther tube of the marginal flower protrudes first, followed by a flower in the middle. Simultaneously, the marginal flower corollas open, with openings happening first on the periphery and in turn towards the center. According to the changes in the characteristics of the *N. insignis* flowers, the opening process of the capitulum can be divided into 6 stages (Figure 2a,b): (a) The top of the conical buds expands and the brown pappi are exposed (duration: ~2 days); (b) the anther tube of the marginal flower protrudes (duration ~2 days); (c) the corollas of the marginal flowers open, the anther tube of the middle flower protrudes, the stigma elongates, and a small amount of pollen is pushed to the top of the anther tube (Figure 2h; duration: ~3 days); (d) the corollas of the middle flowers open, the stigma continues to elongate, a large amount of pollen in the anther tube is pushed to the top of the anther tube, and pollen piles up for export (duration: ~3–4 days); (e) pollen is gradually scattered, the stigma lobes spread a “Y” type into the female stage, and the inside pollination surface is exposed to receive pollen (Figure 2i); and (f) pollination is completed, the corolla lobes adopt a yellowish brown color, and the anther tube and stigma lobes gradually shrink. The whole process shows typical female and male heterotopic characteristics, and it exhibits obvious characteristics of pollen secondary display and protandry.

Flowering phenology of *N. insignis* was observed during the spring periods (February to April) of 2014–2020 in Yuanmou County within the dry and hot valley of the Jinsha River, southwest China. As the capitulum of the Asteraceae plant is considered to be a single flower, the observation of flowering dynamics is generally carried out for the whole capitulum (Burtt, 1961; Mani & Saravanan, 1999). According to Burtt (1961), the blooming of involucral bracts outside the capitulum bloom marks the starting stage to flowering, and the florets opening in the middle of inflorescence marks the end of flowering period. Five phenological parameters were derived from the flowering data: (i) onset (date of first flower); (ii) duration (based on date of first and last flower); (iii) mean peak flowering date; (iv) mean flowering amplitude (number of flowers produced per unit time); and (v) synchrony (flowering overlap among individuals). All these variables of flowering phenology were derived from 8 well‐growing individuals with the diameter at breast height (DBH) larger than 5 cm randomly chosen from the population, which contained more than 60 individuals with DBH larger than 3 cm. The chosen individuals were located in the middle of the population, and the distance between each individual was 10–15 m. We marked these individuals with red paint and the same individuals were used for sampling every year. The method used to quantify floral phenology closely followed McIntosh (2002).

Flowering synchrony within an individual plant indicates the degree to which the blooming period of the plant overlapped the blooming period of all the other plants within the population. Flowering synchrony was calculated using the method of Primack (1980). The index of synchrony (X) for an individual plant was estimated as:

$$X_{i}=\left(\frac{1}{n‐1}\right)\left(\frac{1}{f_{i}}\right)\sum_{j=1}^{n}e_{j\neq i},$$

where *e_j_* is the number of weeks the flowering periods of individual i and j overlapped; *f_i_* is the total flowering period of individual i in weeks, and *n* is the number of individuals in the sample. *X* varies from 1 (plant flowering overlaps with that of all other individuals) to 0 (no overlap with any other individuals).

### 2.3 Floral biology

Floral morphology was observed in the field and also in the laboratory with the help of a stereomicroscope (Leica M80). The pollen viability and stigma receptivity were estimated according to the methods of Dafni (1992) and Nebot et al. (2016). For the determination of pollen grain and ovule numbers, a total of 30 flowers from 8 sampling individuals (3–5 flowers per individual) were randomly chosen just before flower opening and preserved in 70% ethanol. To determine the pollen grain number, dehiscing anthers were placed in tubes filled with 1 ml ethanol and shaken with a vortex mixer to release the pollen; and the number of pollen grains was counted at 8× magnification by a dissecting magnifying glass. The number of pollen grains per tube was extrapolated to obtain the number of pollen grains available per anther. The number of pollen grains per anther was multiplied by number of anthers per flower in order to obtain the number of pollen grains per flower. The ovaries were dissected with a scalpel and placed in a drop of water on a microscope slide. Ovules were counted at 1.5× magnification under a dissecting magnifying glass.

In terms of pollen viability, 12 anthers were randomly selected from different capitula at different developmental stages. According to the TTC (2, 3, 5‐triphenyl tetrazolium chloride) method, the staining of pollen grains was observed under the microscope (×10), and the number of stained pollen grains was counted (×40). The appearance of a red color indicated vitality, and light red, no change, black, or yellow indicated no vitality. Pollen viability was assessed by red pollen staining rate. Stigma receptivity was checked by the benzidine‐hydrogen peroxide method (benzidine: hydrogen peroxide: water = 4:11:22). In the experiment, 12 stigmas were randomly selected from different capitula at different developmental stages and placed on 12 concave glass slides. After dropping a small amount of benzidine‐hydrogen peroxide reaction solution, cover the glass slide and observe the receptivity of the stigmas under the optical microscope (×40). The appearance of a blue color and a large number of bubbles indicates receptivity.

### 2.4 Visit frequency and behavior of pollinators

To clarify the temporal variation of pollinator activity, the frequency and types of pollinators visiting *N. insignis* flowers were assessed in the spring periods of 2014–2020. During the peak flowering period, counting of the visiting insect species and their frequency was done on all the eight chosen trees. The observations were made on ten randomly chosen adjacent flowers (capitulum) per tree. The species of flower visiting insects, the duration of each visit, contact with the reproductive parts, and interactions with other visitors were recorded through direct observation and with the help of video cameras. We selected five sunny days (observe 1–2 trees each day) and observed the whole day between 0800 hr and 1800 hr, with each observation period lasted about 15 min and each observation interval of 1 hr. The frequency of pollinators was assessed in terms of visits/flower/hour. All visitors were video recorded and collected. These insects were then identified by an entomologist.

### 2.5 Pollination experiments

Based on randomly selected 30–40 capitula each year, we have investigated the natural seed setting rate of *N. insignis* for 7 consecutive years (from 2014 to 2020). To determine the breeding system of *N. insignis* and the contribution of insect visitors to effective pollination, the following treatments were created with 30–40 randomly selected capitula per treatment in 2020; (a) spontaneous autogamy, in which capitulum buds were bagged with a fine nylon mesh net to exclude insect interactions; (b) obligated autogamy, in which capitulum buds were pollinated with their own pollen and bagged with sulfuric acid paper; (c) geitonogamy, in which capitulum buds were pollinated with pollen of capitulum from the same plant and bagged with sulfuric acid paper; (d) xenogamy, in which capitulum buds were pollinated with pollen of capitulum from the other plant and bagged with sulfuric acid paper; (e) supplementary pollination, in which capitulum buds were pollinated with outcross pollen without bagging. The reproductive success of each pollination treatment was compared in terms of seed setting rate, assessed as the proportion of treated flowers that eventually produced seeds.

### 2.6 Data processing and statistical analyses

We analyzed variation in (a) the date of the first and final flowering between years, (b) the flowering synchrony indices between years, (c) the duration of pollen vitality and stigma receptivity between years, and (d) seed production in different experiment treatments with an analysis of variance (ANOVA) followed by Tukey's studentized range (HSD) test. In terms of the frequency of pollinators visiting flowers, independent‐sample *t*‐test was performed to compare the number of flower visits per capitulum among peak flowering stage and early flowering stage, and among peak flowering stage and late flowering stage. To explore the correlation between seed setting rate and parameters of reproductive characteristics, and between flowering time and rainfall before flowering, we used the method of general linear regression model. The rainfall data were provided by Desert Ecosystem Research Station in Yuanmou County of Yunnan Province, State Forestry Administration of China, and 6 km away from the study area. Analyses were conducted using SPSS 16.0 (IBM; Armonk, NY).

## 3 RESULTS

### 3.1 Flowering phenology

**FIGURE 3:**
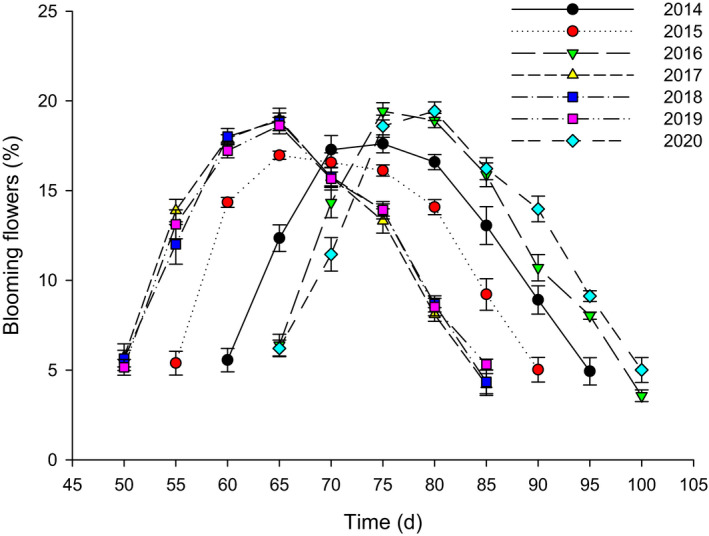
Flowering phenology characteristics of *Nouelia insignis*. Dates are given by day of the year, with 1st January = 1

The flowering duration of the population of *N. insignis* lasted about 35 days and without significant differences between years (Figure 3). However, significant differences were apparent in the date of the first flowering and the date of the final flowering between years (*F* = 63.653, *df* = 6, *p* < .01; *F* = 60.821, *df* = 6, *p* < .01). Flowering dates in 2017, 2018, and 2019 were obviously earlier than those in 2014, 2015, 2016, and 2020, and the earliest flowering dates (such as in 2017, 2018, and 2019) were on average 15 days earlier than the latest flowering dates (such as in 2016 and 2020).

The flowering synchrony indices of 2014, 2015, 2016, 2017, 2018, 2019, and 2020 were 0.89, 0.85, 0.79, 0.82, 0.80, 0.81, and 0.80, respectively, and the results of variance analysis showed that there were no significant differences in flowering synchrony for different years (*F* = 0.191, *df *= 6, *p* > .05).

### 3.2 Floral biology

**FIGURE 4:**
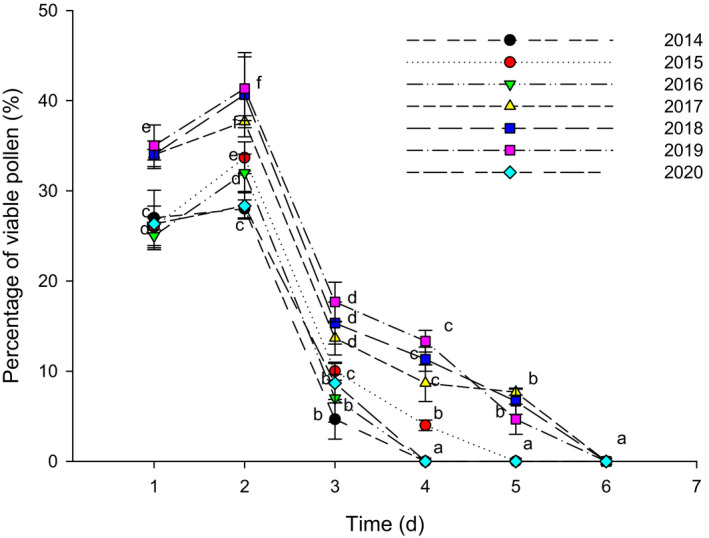
Changes of pollen vitality after pollen being pushed out from anther canister. Different letters in the same year indicate significant differences (*p* < .05)

All florets were protandrous, and the mature pollen dispersed in the anther canister. The pollen grains had a strong vitality when they were pushed out from the anther canister by stigmata, and the highest pollen vitality was reached on the second day, and then, it has dropped significantly until it reached zero (Figure 4). The pollen usually could keep its vitality for 3–5 days, and significant differences were found among different years. Specifically, it lasted for 3 days in 2014, 2016 and 2020, and 4 days in 2015, and 5 days in 2017, 2018, and 2019.

**FIGURE 5:**
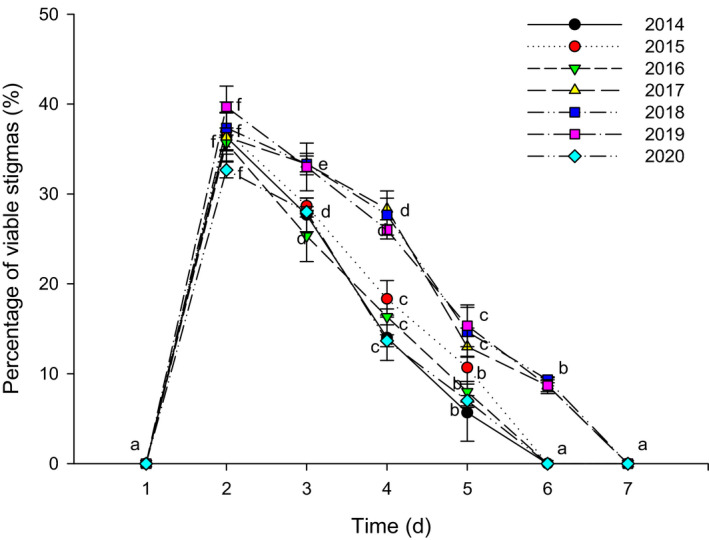
Changes of stigma receptivity after stigma stretching out from anther canister. Different letters in the same year indicate significant differences (*p* < .05)

In the early stage of the stigmata protruding from the anther canister, the stigmata lobes were unopened, and the stigmata were not receptive at this time. During the second day, the stigma lobes showed a Y‐shaped pattern of separation, and the stigma had the greatest receptivity (Figure 5). This indicated that the best time for pollination was on the second day when the stigmata protruded from the anther canister. From the third day, the receptivity of the stigma declined rapidly until it reached zero. The stigma was keep receptive for 4–5 days, and significant differences were found among different years. Specifically, it lasted for 4 days in 2014, 2015, 2016, and 2020, and 5 days in 2017, 2018, and 2019.

### 3.3 Seed production

**FIGURE 6:**
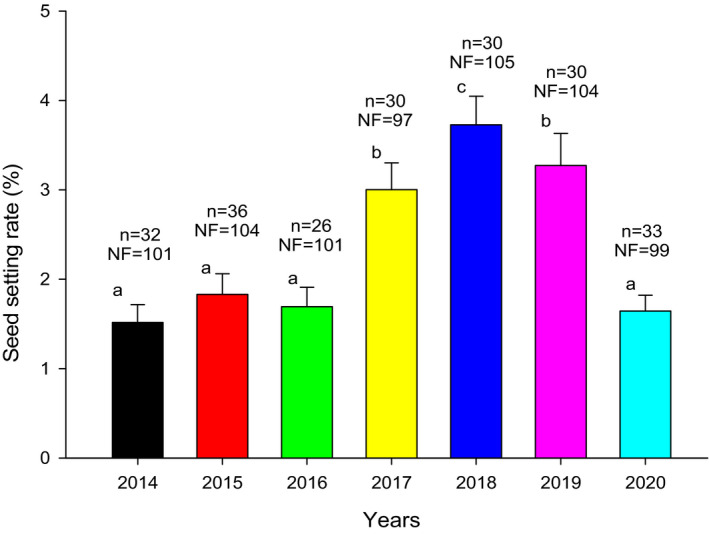
Seed setting rate of *Nouelia insignis* under the natural pollination condition. n: sampling quantity; NF: number of florets in each capitulum; different letters between bars indicate significant differences (*p* < .05)

**FIGURE 7:**
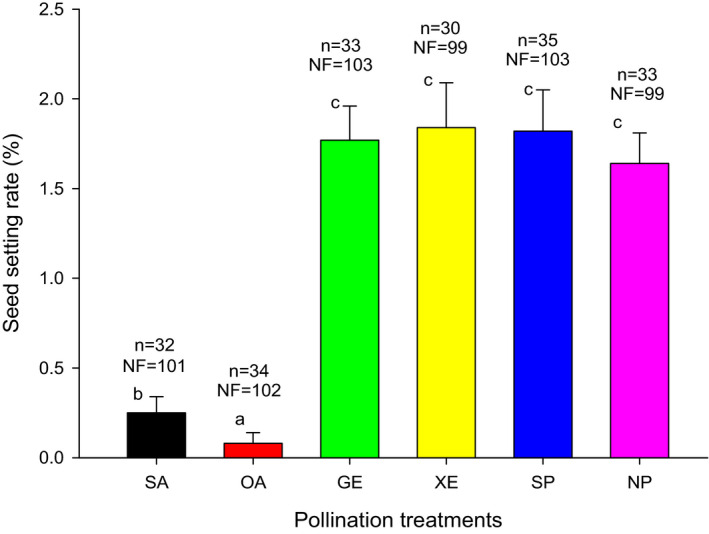
Comparison of seed setting rate of *Nouelia insignis* between different pollination treatments. SA: spontaneous autogamy, in which capitulum buds were bagged with a fine nylon mesh net to exclude insect interactions; OA: obligated autogamy, in which capitulum buds were pollinated with their own pollen and bagged with sulfuric acid paper; GE: geitonogamy, in which capitulum buds were pollinated with pollen of capitulum from the same plant and bagged with sulfuric acid paper; XE: xenogamy, in which capitulum buds were pollinated with pollen of capitulum from the other plant and bagged with sulfuric acid paper; SP: supplementary pollination, in which capitulum buds were pollinated with outcross pollen without bagging; NP: natural pollination (2020), in which capitulum buds were not manipulated. n: sampling quantity; NF: number of florets in each capitulum; different letters between bars indicate significant differences (*p* < .05)

There was a significant difference in seed setting rate under the natural pollination condition across different years (*F* = 7.534, *df* = 6, *p* < .01). The highest seed setting rate was 3.73% in 2018, and the lowest was 1.52% in 2014 (Figure 6), which showed that the seed setting rate might be affected by the environmental changes in different years. The seed setting rates of geitonogamy treatments (GE), xenogamy treatments (XE), and supplementary pollination treatments (SP) were 1.77%, 1.84%, and 1.82%, respectively, which were slightly higher than those of the natural pollination treatments (NP) (1.64%) (Figure 7). However, no significant differences were found between them (*p* > .05). Obligated autogamy treatments (OA) and spontaneous autogamy treatments (SA) had less seed formation, with seed setting rates of 0.08% and 0.25%, respectively, which were significantly lower than those of other treatments (*p* < .05). Significant differences in seed setting rate were observed between obligated autogamy treatments and spontaneous autogamy treatments (*p* < .05).

### 3.4 Insect visitors and their behavior

**FIGURE 8:**
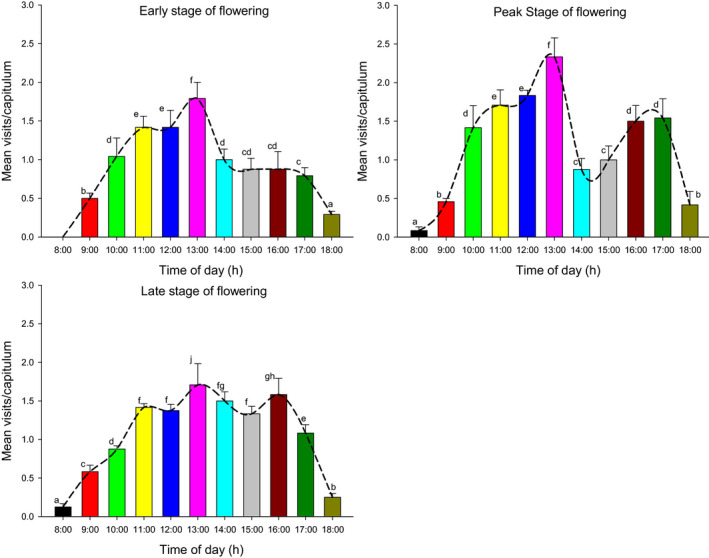
Visit frequencies of insects during flowering period of *Nouelia insignis* (n/h per capitulum). Different letters between bars indicate significant differences (*p* < .05)

During the observation period, seven species of flower visitors were observed for *N. insignis*. These were as follows: *Rhopalomelissa yasumatsui*, *Stomorhina lunata*, *Episyrphus balteatus*, *Apis cerana*, *Sycanus croceus*, *Delta conoideum*, and *Chrysomyia megacephaia*. The visits to the flowers occurred mainly between 8:00 and 18:00, and there were higher frequencies of visits at approximately 13:00 and during 16:00–17:00. Insects had the highest frequency of visiting flowers at the peak flowering stage (2.7/h per capitulum), followed at the late flowering stage (2.3/h per capitulum), and the lowest frequency happened during the early flowering stage (2.2/h per capitulum; Figure 8). The number of flower visits per capitulum at the peak flowering stage was significantly higher than that at the early flowering stage or at the late flowering stage (*T* = 8.859, *p* < .01; *T* = 4.049, *p* < .01).

**FIGURE 9:**
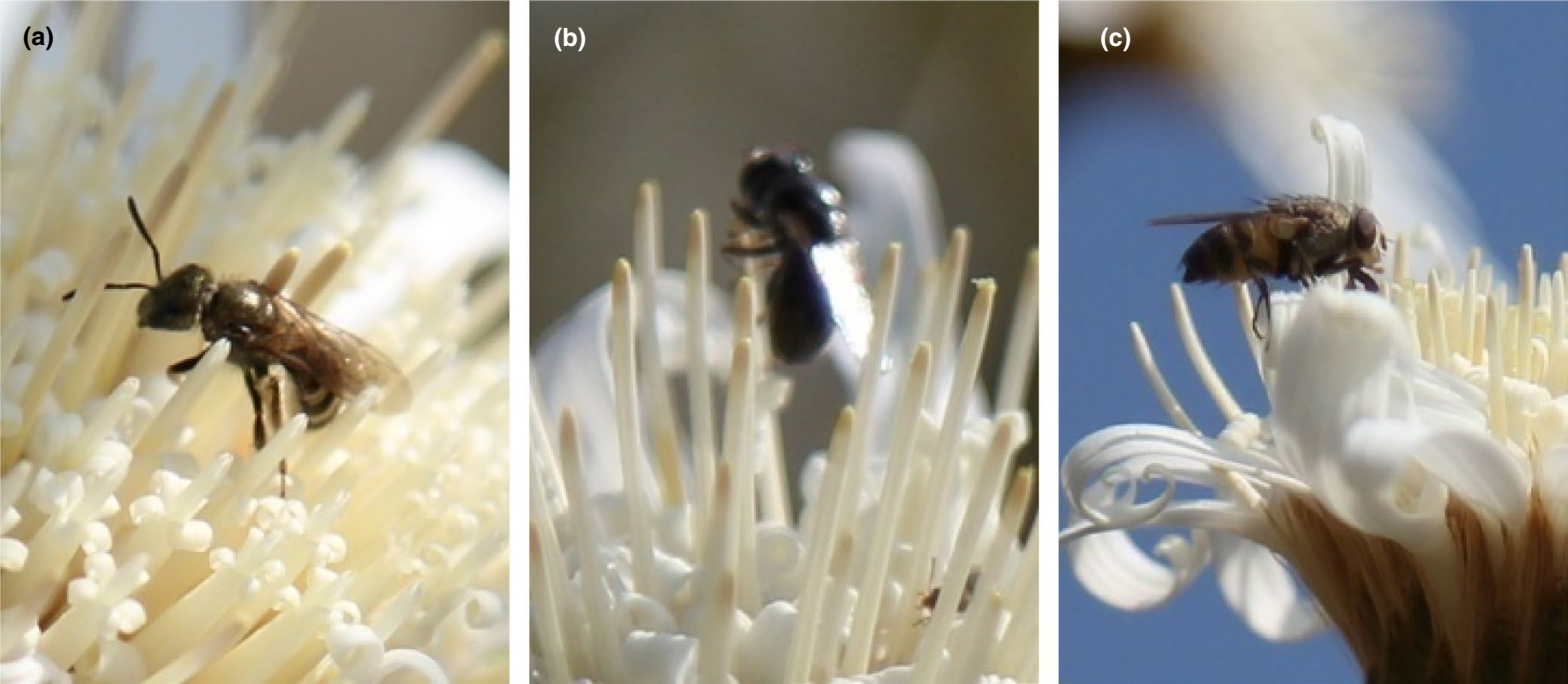
Main pollinators of *Nouelia insignis*

*Apis cerana*, *S. croceus*, *D. conoideum*, and *C. megacephaia* visited flowers less frequently and spent less time per visit (i.e., 3–15 s at each visit of the capitulum). *Rhopalomelissa yasumatsui* (Figure 9a), *S. lunata* (Figure 9b), and *E. balteatus* (Figure 9c) were frequently seen during all periods of observation, and the time they remained on the capitulum at each visit was longer (30–55 s). Among them, *E. balteatus* spent the longest time, up to 55 s for each visit of a capitulum. Field observation found that flower visiting behavior of insects was quite different. *Rhopalomelissa yasumatsui* and *A. cerana* crawled in the floret after flying on the capitulum. When they foraged and crawled, their feet, head, and abdomen could touch the pollen on the surrounding florets, and a large number of pollen was attached to their body hair. *Stomorhina lunata* and *C. megacephaia* would also crawl among the florets. When they foraged for nectar, their forefeet hold the anther tube and licked it with their mouths. In this process, the abdomen and chest could contact the pollen of other florets of the capitulum and became effective pollinators. *Episyrphus balteatus* rarely crawled among the florets, but mainly supported themselves with midfeet and hindfeet, and licked pollen and nectar directly on the anther tube, which resulted in lower pollination efficiency. *Sycanus croceus* and *D. conoideum* often appeared around the capitulum, rarely crawled among florets, and carried less pollen. In addition, there was no obvious distinction in the number and the behavior of flower visitors between years, whether *N. insignis* bloomed early or late.

### 3.5 Factors influencing seed setting rate and flowering time

**FIGURE 10:**
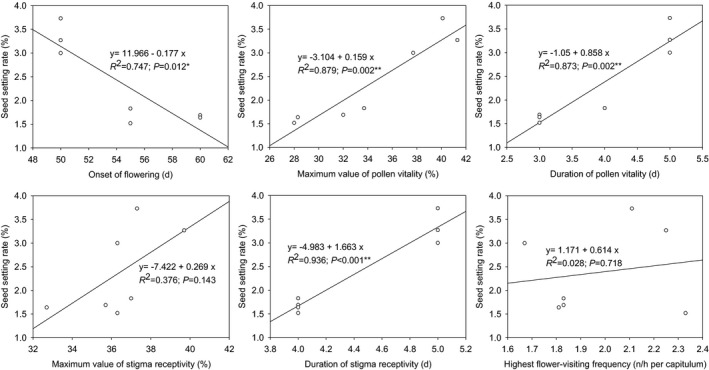
Linear relationships between seed setting rate and parameters of reproductive characteristics of *Nouelia insignis*. y is the seed setting rate; x is the reproductive characteristic parameter; *indicate significant difference at 0.05 level (2‐tailed test); **indicate significant difference at 0.01 level (2‐tailed test)

The regression analysis (Figure 10) showed a significant negative linear relationship between the seed setting rate and the flowering time (*p* < .05). There was a significant positive linear relationship between the seed setting rate and the maximum value of pollen vitality (*p* < .01), the duration of pollen vitality (*p* < .01), and the duration of stigma receptivity (*p* < .01). No significant linear relationship was found between the seed setting rate and the maximum value of stigma receptivity, the highest flower‐visiting frequency (*p* > .05). It was showed that flowering time and characteristics of pollen vitality and stigma receptivity might have significant effects on reproductive success of *N. insignis*, while the visit frequencies of insects might have no significant effect on its reproductive success.

**FIGURE 11:**
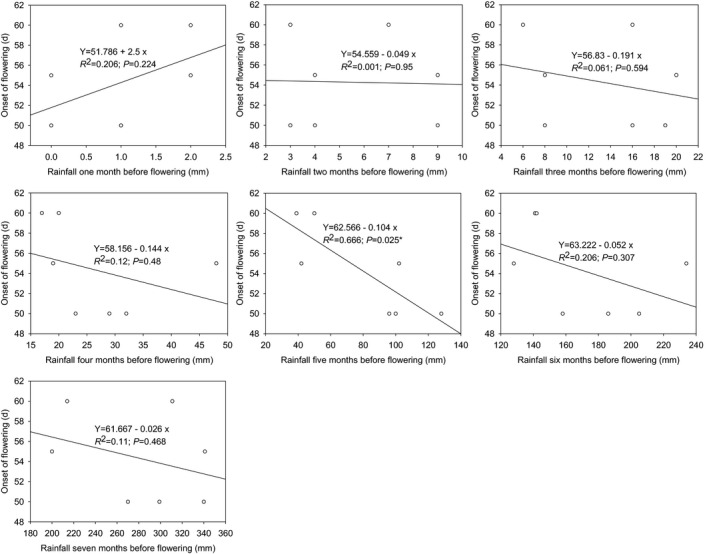
Linear relationships between flowering time and rainfall before flowering of *Nouelia insignis*. y is the flowering time; x is the rainfall before flowering; * indicates significant difference at 0.05 level (2‐tailed test)

The regression analysis showed that there was a significant negative linear relationship between the flowering time and the rainfall 5 months before flowering (*p* < .05; Figure 11 ). No significant linear relationship was found between the flowering time and the rainfall 1, 2, 3, 4, 6, and 7 months before flowering (*p* > .05). It was showed that the rainfall of 5 months before flowering might affect the flowering time of *N. insignis*.

## 4 DISCUSSION

This study demonstrates that there are significant interannual differences in flowering phenology of *N. insignis*. The natural seed setting rate of *N. insignis* in the early flowering year was significantly higher than that in the late flowering year. This may be related to the climatic environment, such as the variability of rainfall before flowering, and not to the behavioral patterns of pollinators.

“Mass‐flowering pattern” or “cornucopia‐flowering pattern” is generally considered as an effective adaptation mechanism to resist adverse environments (Herrerías‐Diego et al., 2006). Due to the flowering synchrony, each plant can exchange genes with most plants of the population, increasing the genetic diversity of the same population (Augspurger, 1981; Martínez‐Sánchez et al., 2011). We found that this flowering pattern also existed in *N. insignis*. Several studies have documented the concentrated flowering patterns of plants helped attract more pollinating insects, which was a kind of reproductive protection to adapt to difficult environment during long‐term evolution (Buide et al., 2002; Herrerías‐Diego et al., 2006), and people have explained many possible reasons for this phenomenon, from plant characters and environmental factors, such as size, flowering duration, and growing season length (Austen et al., 2017). But no significant pollinator‐mediated selection on phenology was detected in some experimental quantified studies (Jiang & Li, 2017). Furthermore, it has been proposed that high seed set was also dependent on high pollinator service, especially for plants that required insect pollination for reproduction (Kudo, 1993; Kudo & Hirao, 2006).

As a matter of fact, there might be significant obstacles to the sexual reproduction process of *N. insignis*. Under natural conditions, the average seed setting rate of *N. insignis* in different years was only 1.52%–3.73%. It was obviously different from other Asteraceae plants that usually had a higher seed setting rate (Grombone‐Guaratini et al., 2004; Hao et al., 2015; Li & Dang, 2007). In our research, insect activities might play a very important role in the pollination process of *N. insignis* (Figure 11). However, we did not detect pollinator limitation in *N. insignis*, and *R. yasumatsui*, *A. cerana*, *S. lunata,* and *C. megacephaia* were all effective pollinators. Our study also showed that there was no significant difference in the number and the behavior of flower visitors across years, whether *N. insignis* bloomed early or late, and no significant relevant relationship was found between visit frequencies of insects and seed setting rate (Figure 9). It suggested that lack of the pollinators was not a limitation for reproductive fitness in *N. insignis*, although the number of pollinators was small and the frequency of visits was low. Simultaneously, artificial auxiliary pollination experiments, including experiments of geitonogamy, xenogamy, and supplementary pollination, did not significantly increase the seed setting rate of *N. insignis*, indicating that pollen limitation was not important factor affecting the reproductive success of *N. insignis* (Figure 11). This study added the evidence of the relationship between reproductive fitness and insect activity, as well as the relationship between reproductive fitness and pollen limitation.

Floral biology in *N. insignis* was detected significant differences among different years (Figures 4 and 5), and a significant linear relationship between some parameters of floral biology such as pollen vitality and stigma receptivity and seed setting rate was observed (Figure 9). This result was consistent with what was proposed in previous studies, in which it was believed that the duration of pollen vitality and stigma receptivity directly affected the seed setting rate (Mani & Saravanan, 1999; Wyatt, 1983). Furthermore, we found that the pollen vitality of *N. insignis* was always low (41.3% at the highest), and the pollen maintained its vitality for a very short period (only 3–5 days). At the same time, the highest proportion of stigmas with vitality was only 39.7%, and the stigma kept receptivity for 4–5 days. In Asteraceae plants with reported breeding systems, pollen activity reached up to 90%. For example, *B. pilosa* reached 98.83%, *E. breviscapus* reached 95%, and *C. lanceolata* reached 94% for pollen vitality. Even on the day before the flower faded, *C. lanceolata* pollen vitality remained around 80% (Grombone‐Guaratini et al., 2004; Li & Dang, 2007; Zeng et al., 2010). The pollen vitality of *A. artemisiifolia* and *S. canadensis* was relatively low, but they also reached 56.8% and 67.0%, respectively. In addition, their pollen vitality was maintained for 8 days and 7 days, respectively. In terms of stigma receptivity, the active stigma of *A. artemisiifolia* reached up to 55.6%, and the viability was maintained for about 12 days. The stigma receptivity of *S. canadensis* was above 50.0%, and the viability was maintained for about 10 days (Hao et al., 2015; Li & Dang, 2007). Compared with other Asteraceae plants, the pollen vitality and stigma receptivity of *N. insignis* were relatively low, and the pollen vitality was maintained for a very short time, which would limit the success of pollination, resulting in the inability to produce a large number of effective seeds.

Flowering time is explained mainly by environmental variation, and it has been proposed that flowering time is a plastic trait that responds to various environmental cues (Lessard‐Therrien et al., 2013; Silva et al., 2011). The plasticity of plant reproductive phenology, especially the flowering time, reflects the adaptability of plant reproduction to environmental changes (Davis et al., 2010; Matthews & Mazer, 2016; Rathcke & Lacey, 1985; Siegmund et al., 2016). For example, in seasonally arid areas, water is a temporally limited resource, and this seasonal variation is one of the most important abiotic factors influencing flowering time (Bullock, 1995; van Schaik et al., 1993). The present study demonstrated that *N. insignis* had big fluctuations in flowering time across years (Figure 3), and flowering time was significantly related to the seed setting rate (Figure 9). Further researches showed that there was a significant linear relationship between flowering time and rainfall 5 months before flowering (Figure 10). This was inconsistent with what has been documented in other seasonally dry tropical forests, where the flowering time depended on the first heavy rains following the dry season (Domínguez & dirzo, 1995; Lampe et al., 1992), and suggested that the rainfall from the end of the rainy season to the middle of the dry season (from October to February of the following year) had a great influence on the flowering time of *N. insignis*. This result might be explained by considering that flowering time was affected by regional environmental variation (Lessard‐Therrien et al., 2013; Silva et al., 2011), because there was almost no heavy rainfall, or even very little rainfall, 2–3 months before flowering of *N. insignis* in the dry‐hot valley. This observation provided the first description of the relationship between flowering time of *N. insignis* and rainfall before flowering.

## 5 CONCLUSION

Our results indicate that the seed setting rate of *N. insignis* is low in the natural condition and varies greatly from year to year. Neither pollinator limitation nor pollen limitation causes the low seed setting rate of *N. insignis*. The obstacles in sexual reproduction process of this species may be attributable to its low stamen and pistil functions, such as poor pollen viability and stigma receptivity. The flowering time of *N. insignis* depends on the rainfall 5 months before flowering, which has a significant effect on seed setting rate. In addition, our study represents a relatively short period of time, especially in terms of factors affecting seed setting rate and flowering time, and it is uncertain whether our results will differ in a longer time series. Consequently, long‐term studies are necessary, including other phases of the reproductive cycle such as seed germination, analyzed from the functional and phylogenetic perspectives.

## CONFLICT OF INTEREST

None declared.

## AUTHOR CONTRIBUTIONS

**FangYan Liu:** Funding acquisition (equal); investigation (equal); methodology (equal); writing–original draft (equal); writing–review and editing (equal). **Chengjie Gao:** Funding acquisition (equal); investigation (equal); methodology (equal). **Min Chen:** Investigation (equal); methodology (equal); writing–original draft (equal). **Guoyong Tang:** Investigation (equal). **YongYu Sun:** Investigation (equal); methodology (equal). **Kun Li:** Funding acquisition (equal); methodology (equal); supervision (equal).

## Data Availability

Data used for the analysis are uploaded in a Dryad repository (https://doi.org/10.5061/dryad.fbg79cnv2).
